# The Pleistocene high-elevation environments between 2.02 and 0.6 Ma at Melka Kunture (Upper Awash Valley, Ethiopia) based upon stable isotope analysis

**DOI:** 10.1038/s41598-024-56768-x

**Published:** 2024-03-19

**Authors:** Giuseppe Briatico, Hervé Bocherens, Denis Geraads, Rita T. Melis, Margherita Mussi

**Affiliations:** 1 Dipartimento Di Scienze Dell’Antichità, Sapienza Università Di Roma, Piazzale Aldo Moro 5, 00185 Rome, Italy; 2 Department of Geosciences, Eberhard Karls University of Tübingen, Hölderlinstrasse 12, 72074 Tübingen, Germany; 3 Italo-Spanish Archaeological Mission at Melka Kunture and Balchit, Melka Kunture, Ethiopia; 4 Senckenberg Centre for Human Evolution and Palaeoenvironment, Sigwartstrasse 10, 72076 Tübingen, Germany; 5 CR2P, Muséum National d’Histoire Naturelle, CNRS, Sorbonne Université, CP 38, 8 Rue Buffon, 75231 Paris cedex 05, France; 6 Dipartimento Di Scienze Chimiche E Geologiche, Università Di Cagliari, 09042 Cittadella Di Monserrato, Italy; 7 CNR-IGAG, Piazzale Aldo Moro 7, 00185 Rome, Italy; 8 ISMEO, Corso Vittorio Emanuele II 244, 00186 Rome, Italy

**Keywords:** Ecology, Evolution, Biogeochemistry

## Abstract

Pleistocene environments are among the most studied issues in paleoecology and human evolution research in eastern Africa. Many data have been recorded from archaeological sites located at low and medium elevations (≤ 1500 m), whereas few contexts are known at 2000 m and above. Here, we present a substantial isotopic study from Melka Kunture, a complex of prehistoric sites located at 2000—2200 m above sea level in the central Ethiopian highlands. We analyzed the stable carbon and oxygen isotopic composition of 308 faunal tooth enamel samples from sites dated between 2.02 and 0.6 Ma to investigate the animal diets and habitats. The carbon isotopic results indicate that the analyzed taxa had C_4_-dominated and mixed C_3_-C_4_ diets with no significant diachronic changes in feeding behavior with time. This is consistent with faunal and phytolith analyses, which suggested environments characterized by open grasslands (with both C_3_ and C_4_ grasses), patches of bushes and thickets, and aquatic vegetation. However, palynological data previously documented mountain forests, woodlands, and high-elevation grasslands. Additionally, the carbon isotopic comparison with other eastern African localities shows that differences in elevation did not influence animal feeding strategies and habitat partitioning, even though plant species vary according to altitudinal gradients. In contrast, the oxygen isotopic comparison suggests significant differences consistent with the altitude effect. Our approach allows us to detect diverse aspects of animal behavior, habitat, and vegetation that should be considered when reconstructing past environments.

## Introduction

Over the past decades, intensive paleoanthropological research has shown that the fossil record in eastern Africa preserves key information on the early stages of the evolutionary history of hominins^1^. Diverse hypotheses have been developed to explain why and how the evolution of mammals is linked to climatic and environmental changes throughout the Pleistocene^2–8^. These hypotheses are based on the current understanding of the global paleoclimate records, as well as on the development of the East Africa Rift System, where tectonic events have generated significant regional variations in climate, hydrological landscape, and distribution of vegetation^9–11^.

The paleoenvironmental archives are mostly from Pleistocene archaeological and paleontological sites in eastern Africa at low and medium elevations (≤ 1500 m a.s.l.) (e.g., Olduvai Gorge, Lake Turkana Basin, Busidima Formation)^12–19^, whereas few archaeological sites are known at higher elevations (≥ 2000 m a.s.l.), such as Melka Kunture (hereafter MK)^20–24^, Melka Wakena^25–28^, Gadeb^29^, Fanta^30^, and Mount Dendi^31^ in Ethiopia; and Kilombe^32^ in Kenya. Among the Ethiopian sites at high elevations, MK yields a long geo-archaeological sequence spanning from 2.02 Ma to the Holocene^22,33–37^, where the archaeological layers that recorded fauna and hominin remains, pollen, phytoliths, and ichnological evidence are coupled with accurate stratigraphic positioning.

The present study discusses a comprehensive stable isotopic composition of carbon and oxygen in 308 faunal tooth enamel samples from MK at sites dated between 2.02 and 0.6 Ma. We apply the isotopic data to provide information on the diet and habitat of the Pleistocene fauna and evaluate possible dietary variability over time. Furthermore, we integrate our interpretation with other proxies (e.g., faunal taxonomy, pollen, and phytolith data) to better characterize the environmental conditions of the area. Finally, we compare our isotopic results with other published data from eastern African sites at low and medium elevations to evaluate whether the differences in altitude and vegetation composition influenced animal feeding strategies and behavior. This is the first substantial stable isotopic study along the MK archaeological sequence.

### Archeological background

**Figure 1:**
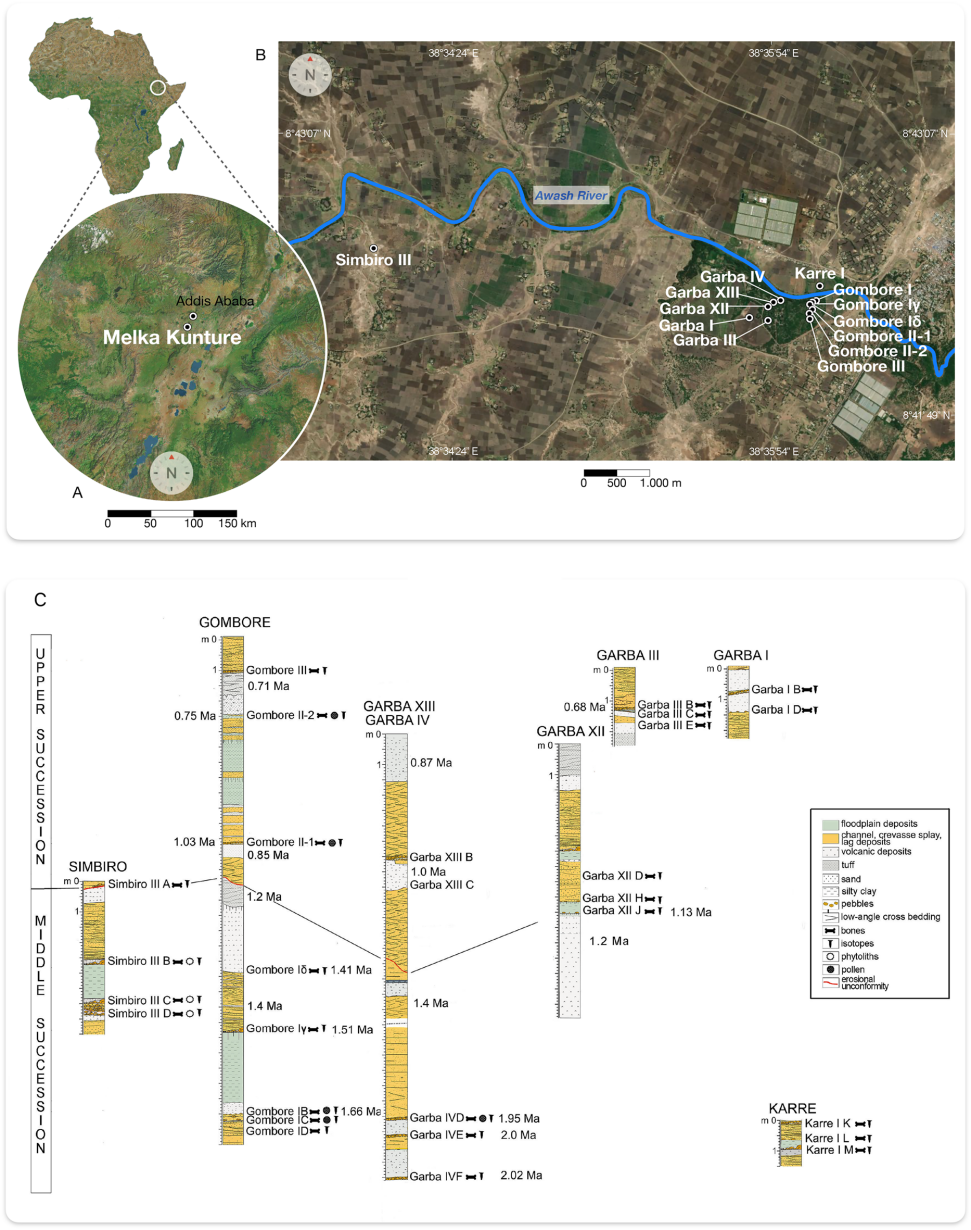
(**A**) Location of MK in the Upper Awash Valley of Ethiopia; (**B**) map of the archaeological sites involved in this study (Apple Maps version 3.0 – 2811.22.9.28.12); (**C)** MK stratigraphic sequences: the sedimentary interpretation of Garba, Gombore, and Simbiro is from one of us (R.T.M.), whereas the succession from Karre is according to Chavaillon and Piperno^20^. Chronology is from Morgan et al.^33^ and Perini et al.^35^.

MK is a cluster of prehistoric sites located approximately 50 km southwest of Addis Ababa, on the western shoulder of the Main Ethiopian Rift, between 2000 and 2200 m a. s. l.^20–22^, extending over 100 km^2^ on the banks of the Upper Awash River (Fig. 1A, B). The stratigraphic sequence was controlled by the interplay between variable tectonic, fluvial, and volcanic activities placed in a low-energy floodplain^38–41^. The archaeological sequence begins with the Early Stone Age, with Oldowan lithic tools (2.02 Ma) and Early, middle, and final Acheulean artifacts (1.95–0.6 Ma)^23,24,35,36,42,43^. The Early Middle Stone Age is documented at ~ 200 ka, whereas the Late Stone Age, mainly found in surface dispersions, is now investigated in a stratified deposit dated to the Holocene^22^. Fossil remains of *Homo erectus*, of a hominin related to *H*. *heidelbergensis*, and of an archaic *H*. *sapiens* were discovered within clear stratigraphic contexts and are directly associated with lithic technocomplexes and faunal remains^24,44–47^. The faunal assemblage is dominated by *Hippopotamus* cf. *amphibius*, a large-sized hippo similar to the modern one. In contrast, the dwarf hippo (*Hippopotamus* cf. *aethiopicus*) is less represented. Alcelaphini is the most common bovid tribe recorded so far, whereas a few specimens refer to buffaloes (Bovini), kobs (Reduncini), and gazelles (Antilopini). The development of endemic mammal sub-species (e.g., *Connochaetes gentry leptoceras* and *Damaliscus strepsiceras*) points to a relative degree of isolation in the highlands due to the topographic relief. Equids are represented by the bones and teeth of *Equus* and *Hipparion* s.l., but the taxonomic identification was difficult. Suids are quite rare, with remains of *Kolpochoerus*, *Metridiochoerus*, and *Phacochoerus* sp. For giraffids, the record includes *Giraffa* sp. and the short-limbed *Sivatherium maurusium*. A few remains of elephants, rhinos, and crocodiles are recorded. The baboon *Theropithecus* cf. *oswaldi* is the only non-human primate attested. Carnivores are extremely rare, consisting of a few remains of *Pseudocivetta ingens*, *Megantereon*, *Lupulella*, and *Crocuta* sp. In addition, two rodent species have been identified as *Oenomys kunturensis* and *Tachyoryctes konjitae*^48,49^ (Tab. S1). Mammals, including hominins, are also attested by footprints in many Pleistocene horizons within the Gombore gully^50–52^.

All over the MK sequence, the vegetation was of the Dry evergreen Afromontane Forest and Grassland Complex (DAF) type^53^, ranging from forest to grassland and bushland, with variations in the distribution of trees, grasses, and herbs over time (Tab. S2; Fig. S1). The DAF vegetation currently characterizes the higher mountain ranges of eastern Africa with a cool and rainy climate. The plant species greatly differs from the warmer and drier plant and tree species of the African savanna at lower elevations^53,54^. In Ethiopia, the DAF vegetation is found between 1800 and 3000 m a.s.l. In the MK area, where it would still develop, it has disappeared in modern times due to anthropic impact^54,55^.

The modern rainfall distribution is bimodal, influenced by the Indian Monsoon and the seasonal oscillation of the Inter-Tropical Convergence Zone (ITCZ). During a short rainy season (locally named *Belg*) from March to June and long summer rains (locally named *Kiremt*) from July to October, the wind direction changed from the northeast to southeast, which brought moisture from the Southern Indian Ocean. The dry season (locally named *Bega*) from November to February begins when the north-easterly trade winds from the Arabian Sea prevail^56^. According to the Ethiopian Meteorological Institute^57^, in the Upper Awash Valley at ~ 2000 m and above, 1000 mm of total rainfall was recorded in 2022, with mean maximum and minimum temperatures of 26 °C and 11 °C, respectively (Fig. S2).

## Results

We report on *δ*^13^C and *δ*^18^O values of six mammalian families (Hippopotamidae, Bovidae, Equidae, Suidae, Hyaenidae, and Giraffidae) in order of their specimen abundance and combined with each archaeological locality, stratigraphic level, and chronology (Fig. 1C; Tab. S3). Overall, 308 isotopic results are reported with median and average values and ranges for each mammalian family. The sampling strategy reflects relative faunal abundance. Indeed, Hippopotamidae and Bovidae are the most abundant taxa in faunal assemblage and isotopic datasets.

### Hippopotamidae

**Figure 2:**
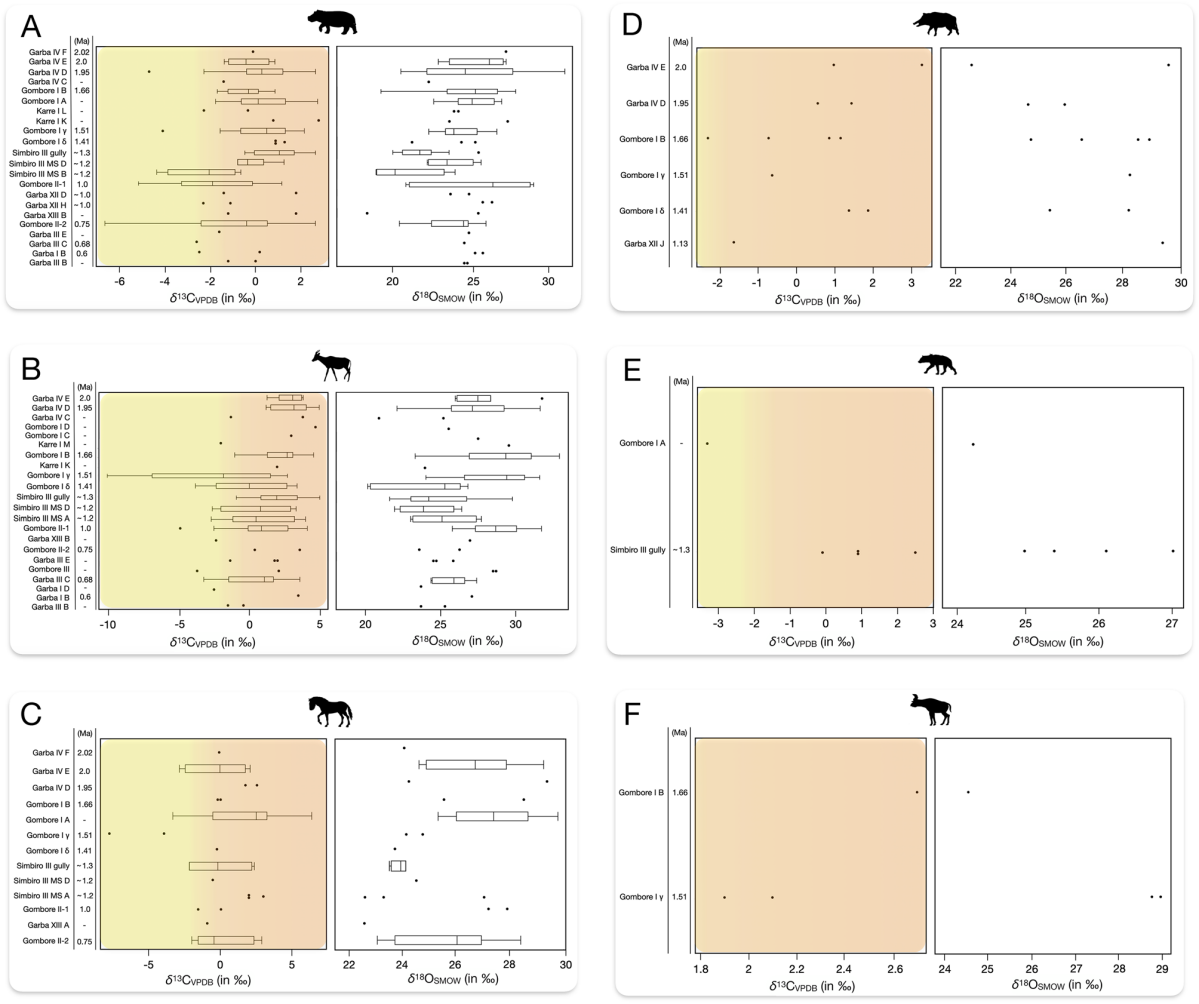
Box and whisker plots of *δ*^13^C and *δ*^18^O values of hippos (**A**), bovids (**B**), equids (**C**), suids (**D**), hyenas (**E**), and giraffids (**F**) from the MK archaeological sites (2.02 – 0.6 Ma). Isotopic data are plotted at the family level. Further details on Bovidae are provided in Fig. S3 (Supplementary Information). The vertical line in the boxes marks the median values; the box ends are the lower and upper quartiles; the lines define the range of data; a solid circle is equivalent to a value. Yellow and orange shades indicate mixed C_3_-C_4_ and C_4_-dominated diets, respectively^16,58^. Animal silhouettes are from Phylopic (https://www.phylopic.org).

The median *δ*^13^C value of *Hippopotamus* cf. *amphibius* (n = 138) was—0.07 ‰, ranging from—6.6 ‰ to + 2.8 ‰. The *δ*^18^O values ranged from + 18.2 ‰ to + 30.9 ‰ with a median value of + 24.3 ‰ (Fig. 2A). The statistics indicate a non-normal distribution of *δ*^13^C values (Shapiro–Wilk Test: *p* = 0.0001), whereas *δ*^18^O values follow a normal distribution (Shapiro–Wilk Test: *p* = 0.9782), which are also confirmed by visual Kernel density and Normal Quantile Plot observations. Statistically significant differences in hippo *δ*^13^C (Wilcoxon/Kruskal–Wallis Rank-Sum Test: *p* = 0.0036) and *δ*^18^O values (Anova Test: *p* = 0.0064) were observed among the archaeological localities.

### Bovidae

The analyzed bovids (n = 105) comprise five tribes (Alcelaphini, Antilopini, Bovini, Hippotragini, and Reduncini) and others not identified below the family level.

Alcelaphini. Samples from alcelaphin bovids (n = 55) had a median *δ*^13^C value of + 2.9 ‰ with values ranging from—2.2 ‰ to + 5.2 ‰, whereas the median *δ*^18^O value was + 27.5 ‰ with values ranging from + 21 ‰ to + 33.1 ‰ (Fig. S3A).

Antilopini. Two samples of Antilopini had *δ*^13^C values of—9.7 ‰ and—2.8 ‰ (average =—6.2 ‰). The *δ*^18^O values were + 26.5 ‰ and + 30 ‰ (average =  + 28.2 ‰) (Fig. S3B).

Bovini. Bovini samples (n = 5) had *δ*^13^C values ranging from—2.3 ‰ to + 3.8 ‰ (average =  + 0.3 ‰). The *δ*^18^O values ranged from + 26 ‰ to + 31.1 ‰ (average =  + 27.9 ‰) (Fig. S3C).

Hippotragini. Samples of Hippotragini (n = 3) had *δ*^13^C values ranging from—2 ‰ to + 2.1 ‰ (average =—0.3 ‰). The *δ*^18^O values ranged from + 24.4 ‰ to + 29.1 ‰ (average =  + 27 ‰) (Fig. S3D).

Reduncini. Samples of Reduncini (n = 3) had *δ*^13^C values ranging from – 5.5 ‰ to + 1.4 ‰ (average =—1.1 ‰). The *δ*^18^O values ranged from + 24.3 ‰ to + 30.7 ‰ (average =  + 27.1 ‰) (Fig. S3E).

Bovidae sensu lato. This group comprises bovid specimens (n = 37) not identified at the tribe and species levels. The median *δ*^13^C value was + 1.3 ‰ with values ranging from—4.6 ‰ to + 5 ‰, whereas the median *δ*^18^O value was + 25.3 ‰, ranging from + 20.2 ‰ to + 31.7 ‰ (Fig. S3F).

Overall, bovid *δ*^13^C values ranged from—9.7 ‰ to + 5.2 ‰ (median =  + 1.9 ‰) (Fig. 2B). The *δ*^13^C values do not follow a normal distribution (Shapiro–Wilk Test: *p* = 0.0001), and the Wilcoxon/Kruskal–Wallis Rank-Sum Test (*p* = 0.0124) showed differences in mean *δ*^13^C values over time. Alcelaphini and Bovidae sensu lato showed higher *δ*^13^C values than those of Antilopini, Bovini, Hippotragini, and Reduncini. However, we noted that the small sample size of Antilopini, Bovini, Hippotragini, and Reduncini limited the effectiveness of statistical analysis. Normal Quantile Plot and Shapiro–Wilk Test (*p* = 0.8177) indicate a normal distribution of *δ*^18^O values, with statistically significant differences (Anova Test: *p* = 0.0001) among the archaeological sites and levels.

### Equidae

This group comprises specimens mostly not assigned to mammalian tribes and species, whereas a few samples belong to *Equus* (n = 4) and *Hipparion* (n = 2) sp. The overall median *δ*^13^C value of equid samples (n = 45) was + 0.1 ‰, with values ranging from—7.6 ‰ to + 6.4 ‰. The median *δ*^18^O value was + 26.7 ‰, ranging from + 23.1 ‰ to + 31.3 ‰ (Fig. 2C). Both *δ*^13^C and *δ*^18^O values are normally distributed (Shapiro–Wilk Tests: *p* = 0.0798; *p* = 0.1170, respectively). Across the sites, statistically significant variability in *δ*^13^C and *δ*^18^O values was observed (Anova Tests: *p* = 0.0427; *p* = 0.0179, respectively).

### Suidae

This family (n = 12) comprises a few samples of *Metridochoerus* (n = 4), *Kolpochoerus* (n = 2), and other suids (n = 6). Overall, average *δ*^13^C value was + 0.05 ‰, ranging from—2.3 ‰ to + 3.3 ‰, whereas median *δ*^18^O value was + 26.8 ‰, ranging from + 22.6 ‰ to + 29.6 ‰ (Fig. 2D).

### Hyaenidae

Five samples of hyena had an average *δ*^13^C value of + 0.1 ‰ (range between—3.3 ‰ and + 2.5 ‰), whereas average *δ*^18^O value was + 25.5 ‰, ranging from + 24.3 ‰ to + 27 ‰ (Fig. 2E).

### Giraffidae

Three samples of giraffids show an average *δ*^13^C value of + 2.2 ‰ (range between + 1.9 ‰ and + 2.7 ‰). The average *δ*^18^O value was + 27.4 ‰, ranging from + 24.5 ‰ to + 29 ‰ (Fig. 2F).

Due to the limited number of Suidae, Giraffidae, and Hyaenidae samples, a robust statistical evaluation was impossible. Therefore, our interpretation is only a preliminary one.

## Discussion

**Figure 3:**
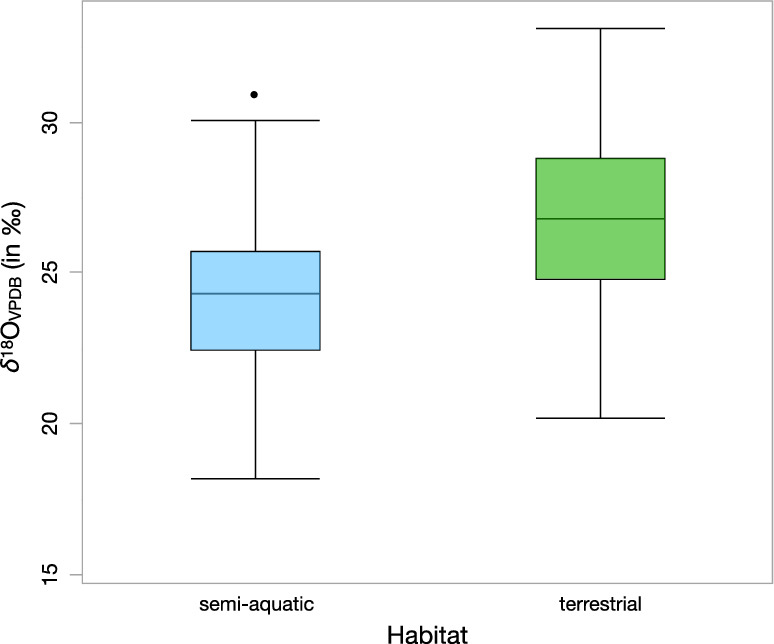
Box and whisker plots of *δ*^18^O values of hippos (semi-aquatic habitat) and bovids, equids, suids, and giraffids (terrestrial habitat). The horizontal line in the boxes marks the median values; the box ends are the lower and upper quartiles; the lines define the range of data; and the solid circle is for outliers.

The *δ*^13^C values suggest that the hippos had a diet dominated by C_4_ forage, although some individuals showed lower *δ*^13^C values, which reflects the opportunistic consumption of C_3_ grasses, fruits, aquatic vegetation, or a combination of these resources^59–65^. Indeed, modern hippos are known to spend most of their daily time close to a body of water, even though they regularly travel some kilometers to forage at night for preferred grass^66^. At MK, there is direct evidence of a 700-ka hippo trail produced by *H*. cf. *amphibius,* which is consistent with modern hippo behavior^50,51^. Bovid and equid *δ*^13^C values indicate that they had C_4_ diets, with some lower *δ*^13^C values interpreted as the outcome of a mixed C_3_-C_4_ diet. In contrast, suids and giraffids had full C_4_ diets, whereas hyena *δ*^13^C values reflect the various isotopic signatures of their prey^67^, which had both C_4_ and mixed C_3_-C_4_ diets (Fig. 2). The consumption of grazers and mixed feeders by hyenas is confirmed by carnivore tooth marks found on a hippo scapula and tibia at Gombore II-2, currently dated to 750 ka^35,50,51^. The *δ*^18^O values of all the taxa from different localities and stratigraphic levels point to variations in the oxygen isotopic composition of animal drinking water sources, food water, and possibly climatic and environmental changes over time. The hippos showed lower *δ*^18^O values than other taxa (e.g., bovid, equid, suid, and giraffid), which is consistent with the expected differences between semiaquatic and terrestrial habitats (Fig. 3), as already evidenced at MK^64,68^ and elsewhere^69,70^. In addition, hippos and hyenas have closely similar median *δ*^18^O values, which seems consistent with our *δ*^13^C values interpretation and previous taphonomic analysis^50,51^.

The isotopic data agree with the analysis of faunal assemblage, suggesting environments characterized by open grasslands, patches of bushes and thickets, and aquatic vegetation. No evidence of pure browsers is attested by either faunal or isotopic analyses^49,64^. This is probably the result of taphonomic issues and the consequent sampling bias. In contrast, hippos are a significant component of the MK paleontological record because of their semi-aquatic habitat, which allows for a greater abundance within fluvial deposits. Caution is required when paleoenvironmental reconstruction is based on limited faunal remains. Pollen and phytolith analyses provide further environmental insights^23,53^. Within the DAF vegetation, which developed throughout the MK archaeological sequence, the proportion of mountain forests, woodlanS1ds, and grasslands was subjected to significant fluctuations^53^. The two periods when mountain grasslands were more extensive than forests are those evidenced at Garba IVD (1.95 Ma) and Garba IB (0.6 Ma) (Fig. S1), which are far from each other in time. Among various sites, there is ample evidence of phases with much more wooded and humid vegetation, with no trend toward increased aridity along the MK sequence. The changes should rather be seen as related to the global glacial and interglacial cycles^53^. The elevation also allowed for the development of diverse environments with C_4_ grass but also C_3_ types of grass, which at MK have been recorded by phytolith analysis^23^, and currently become dominant above 2000 m a. s. l.^71^. This is well-tracked by hippo, bovid, and equid dietary preferences in several archaeological localities with distinct chronologies, such as at Gombore IA (~ 1.6 Ma), Gombore Iγ (1.51 Ma), Simbiro III MS B (~ 1.2 Ma), Gombore II-1 (1.0 Ma), and Gombore II-2 (0.75 Ma). Changes in vegetation composition did not have a major impact on the feeding behavior of mammals, which selected the preferred plant species in the varied mosaic environments. Furthermore, we cannot rule out the possibility that C_3_ isotopic signals are associated with seasonal variations in the proportion of C_3_ and C_4_ grasses reflected in the animal diet. This was demonstrated elsewhere by Souron et al.^63^ using intra-tooth isotopic profiles.

**Figure 4:**
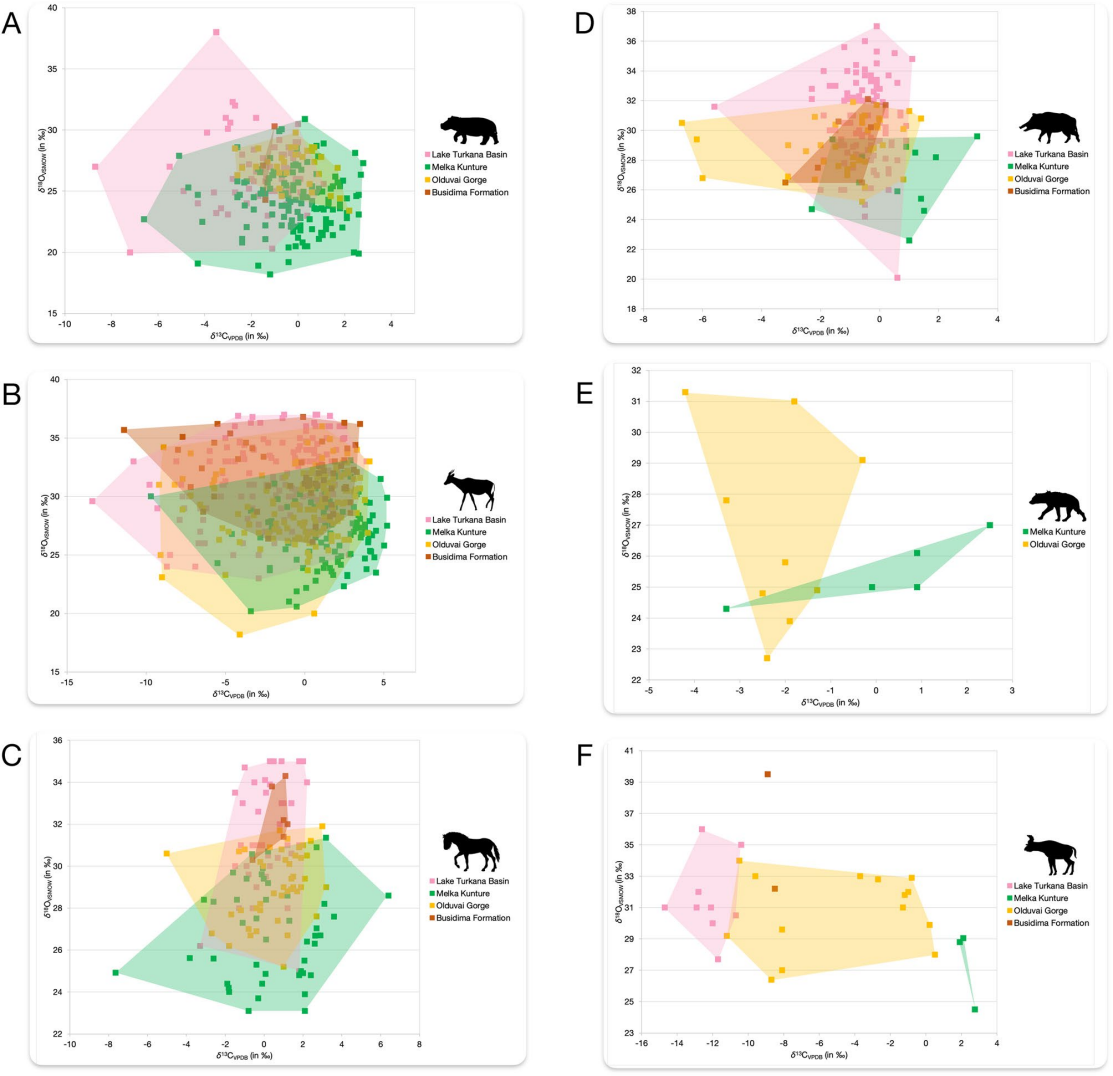
Scatter plots of *δ*^13^C and *δ*^18^O values of hippos (A), bovids (B), equids (C), suids (D), hyenas (E), and giraffids (F) from Lake Turkana Basin, MK, Olduvai Gorge, and Busidima Formation. Animal silhouettes are from Phylopic (https://www.phylopic.org).

In order to test whether the differences in elevation and vegetation influenced the animal feeding strategies and habitats, we compared *δ*^13^C and *δ*^18^O values (n = 1.064) of fauna tooth enamel from published data of eastern African sites at low and medium altitudes (≤ 1500 m a.s.l.), such as Olduvai Gorge^13–16,19^, Lake Turkana Basin^17,65,72^, and Busidima Formation^12^, with isotopic data from the higher elevation site of MK presented in this study. This isotopic dataset includes six faunal families (Hippopotamidae, Bovidae, Equidae, Suidae, Giraffidae, and Hyaenidae) dated between 2.1 and 0.6 Ma (Tab. S4). The comparison of *δ*^13^C values indicates a high degree of overlap in terms of dietary habits, with average diets dominated by C_4_ and mixed C_3_-C_4_ vegetation (Fig. 4). The only exception is represented by some bovids (Tragelaphini and Antilopini) and giraffids (*Giraffa* s.l., *G*. cf. *stillei*, *G*. cf. *jumae*) from the Lake Turkana Basin, Olduvai Gorge, and Busidima Formation, which only consumed C_3_ vegetation (Fig. S5). The median *δ*^13^C value of MK fauna is higher than those of other sites, which suggests a more C_4_-dominated open environment at MK than at the other archaeological sites for the sampled chronology. However, the hippos from MK and Olduvai Gorge show *δ*^13^C values close to each other, and the equids from MK and Lake Turkana have equal median *δ*^13^C values (Tab. S5, Fig. S5). In addition, an increase in *δ*^13^C values has been observed for C_3_ plants with altitude^73,74^. Therefore, the end-member *δ*^13^C value of C_3_ vegetation probably increased with altitude and could explain this slight increase of *δ*^13^C values of herbivorous taxa in MK compared to lower altitude sites due to this shift of *δ*^13^C values for C_3_ plants at higher altitude in taxa that consume some C_3_ plants.

Based on carbon isotopic analysis, we argue that differences in elevation did not influence animal feeding strategies and habitat partitioning, even though plant species vary according to altitudinal gradients^71^. Furthermore, we emphasize that carbon isotopic data alone is insufficient to reconstruct the paleoenvironments directly. Indeed, at MK, *δ*^13^C values suggest the presence of extended grasslands in the landscape. However, there is evidence from pollen and phytolith analyses that the local mountain environment was characterized by a variable proportion of forests, woodlands, and grasslands (with both C_3_ and C_4_ grasses)^23,53^ through time.

Comparing *δ*^18^O values, we observe that taxa from MK had lower *δ*^18^O values than those from the other localities (Tab. S6, Fig. S6), consistent with the altitude effect ^57,75–77^. This is attributed to the progressive condensation of atmospheric vapor and rainout along the mountain slopes and its cool-off, with a consequent loss of H_2_^18^O in the form of rain from cloud moisture. As a result, high-altitude precipitation shows lower *δ*^18^O values than low-altitude precipitation^57^. The altitude effect is reinforced by the difference in temperatures between low- and high-elevation localities^76–78^. Thus, when interpreting *δ*^18^O values from fossil tooth enamel as a paleoclimatic proxy, we should consider that many factors (continentality, source, altitude, temperature, seasonality, and relative humidity) may influence *δ*^18^O variability, making it difficult to establish a clear and direct link with past temperatures^77^.

## Conclusion

Isotopic analyses of fauna tooth enamel from MK provide information on feeding behavior and their adaptation. Overall, between 2.02 and 0.6 Ma, the sampled taxa show a low variability in foraging strategies, which are all within the range of C_4_ and mixed C_3_-C_4_ diets. The DAF vegetation changed over the Lower and Middle Pleistocene times, with variable proportions of mountain plant species^53^, including both C_3_ and C_4_ grasses^23,64^, but this did not influence animal feeding habits. The analyzed mammals were able to select the preferred plants and successfully adapt to the mountain ecosystems. Comparisons with *δ*^13^C values from eastern African sites at low, medium (≤ 1500 m), and high elevations (≥ 2000 m) point to variability in the isotopic signal but not in the animal diets, which is constantly within the range of C_4_ and mixed C_3_-C_4_ diet. The plant species of the African lowlands are different from those at high-elevation^53,71^, but the animal habitat and behavior in selecting food were not influenced along the altitudinal gradient by the changing vegetation.

The *δ*^18^O values recorded significant differences, which were explained as resulting from the altitude effect. The climate of modern-day Ethiopia is influenced by variable altitudes, with mountains up to 4000 m and more, high plateaus between 1300 and 3000 m, and lowlands stretching to sea level. These differences are also reflected in diverse local climates^56,76^.

Between 2.02—0.6 Ma, the human presence at MK is directly recorded by paleoanthropological remains^24,44–46^, as well as by lithic artifacts^23,36,42,43^ and fossil footprints^50–52^. Together with the isotopic results, this allows speculating on human behavior and adaptation. The hominins were able to live and thrive in mountain mosaic ecosystems^24,53,79^, likely exploiting the diverse food resources provided by the DAF vegetation spectrum.

The detailed paleoecological reconstruction is the outcome of the implementation of multiple methodologies, such as stable isotopes, pollen, phytoliths, and faunal taxonomy. Based on our experience with an integrative and complementary approach, caution should be exercised when paleoenvironmental interpretations are determined instead by a single proxy or analyses that are limited by taphonomic bias.

## Material and methods

### Stable isotope analyses

We collected fauna tooth enamel samples (n = 308) from several archaeological localities within the MK complex of sites to analyze the stable carbon and oxygen isotopic compositions. The specimens include Artiodactyla (Hippopotamidae, Bovidae, Suidae, Giraffidae), Perissodactyla (Equidae), and Carnivora (Hyaenidae) in order to represent the full dietary diversity. The enamel samples were collected at the National Museum of Ethiopia (Addis Ababa) in agreement with the Ethiopian Heritage Authority (EHA). Enamel was sampled using a drilling device equipped with a diamond-tipped bit to obtain 12—15 mg of powder. Powdered samples were soaked in 2—3% NaOCl for 24 h at 20 °C to oxidize organic residues and rinsed thrice with Millipore water (Milli-Q H^2^O) to remove all NaOCl. The remaining samples were treated with 0.1 M acetic acid-calcium acetate buffer (pH = 4.66) for 24 h at 20 °C to remove exogenous carbonate. Samples were rinsed thrice with Milli-Q H^2^O and placed in an oven to dry at 40 °C for 72 h. Only 2.5—3 mg of structural carbonate was subjected to Isotopic Ratio Mass Spectrometry (IRMS) at the Biogeology Research Group of the University of Tübingen (Germany). Further details are provided in Supplementary A and B.

### Statistical analysis

Statistical tests were performed using JMP 17 Pro (licensed by Sapienza University of Rome), with the significance level set at *p* = 0.05. The Shapiro–Wilk test was used to verify whether the data followed a normal distribution. When the hypothesis of the normal distribution was accepted, parametric tests such as the t-test and the analysis of variance (ANOVA) test were used to test whether the *δ*^13^C and *δ*^18^O values significantly differed over time for each archaeological site. For the same purpose, non-parametric tests such as the Mann–Whitney U-test (also known as Wilcoxon Rank Sum Test) or the Kruskal–Wallis test were used when the hypothesis of the normal distribution was rejected. The t-test and Mann–Whitney U-test were applied with two groups or levels, whereas ANOVA and Kruskal–Wallis tests were applied with three or more groups.

### Supplementary Information

Supplementary Tables.

Supplementary Figures.

## Data Availability

The data generated and analyzed during the study are available in this published article and its Supplementary Information files.
