# Supplementary material for: The Pleistocene high-elevation environments between 2.02 and 0.6 Ma at Melka Kunture (Upper Awash Valley, Ethiopia) based upon stable isotope analysis
Source: Sci Rep. 2024 Mar 19;14:6619. doi: 10.1038/s41598-024-56768-x (PMC10950861; doi:10.1038/s41598-024-56768-x)
Supplement: Supplementary file 2 — Supplementary Figures. [file 41598_2024_56768_MOESM2_ESM.pdf]

Supplementary Information

The Pleistocene high-elevation environments between 2.02 and 0.6 Ma at Melka Kunture (Upper Awash Valley, Ethiopia) based upon stable isotope analysis

Giuseppe Briatico*, Hervé Bocherens, Denis Geraads, Rita T. Melis, Margherita Mussi

* Corresponding author. E-mail address: giuseppe.bria@gmail.com (G. Briatico).

This PDF file includes:

Figs. S1 – S6
Supplementary A. Stable carbon and oxygen isotopes in tooth enamel
Supplementary B. Laboratory activities
Supplementary C. Isotopic comparison of Early and Middle Pleistocene fauna
References

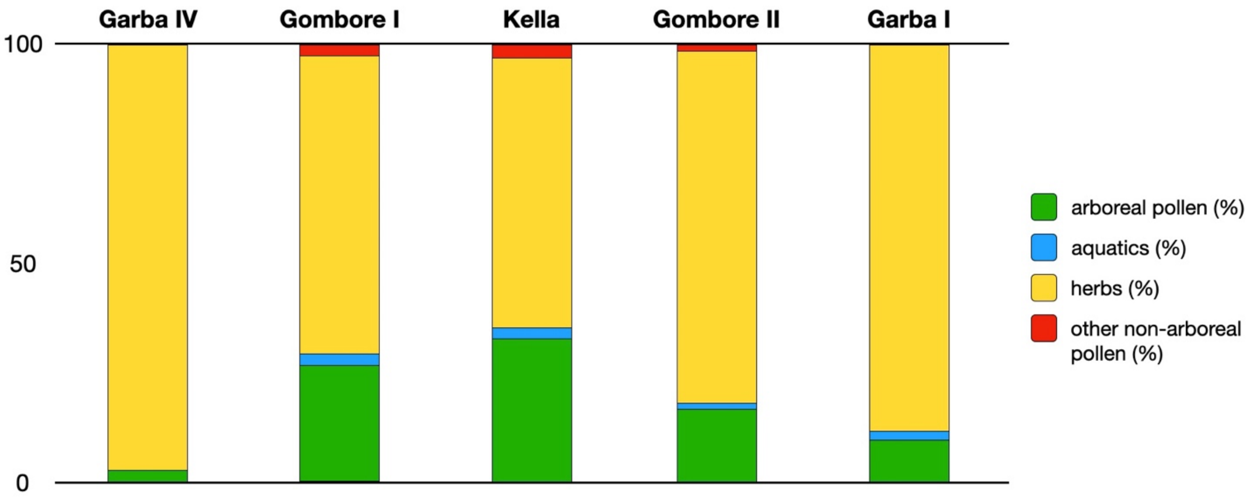

Figure S1. Percentages of the pollen categories in the samples of Table S2 (redrawn from Geraads et al.[1]).

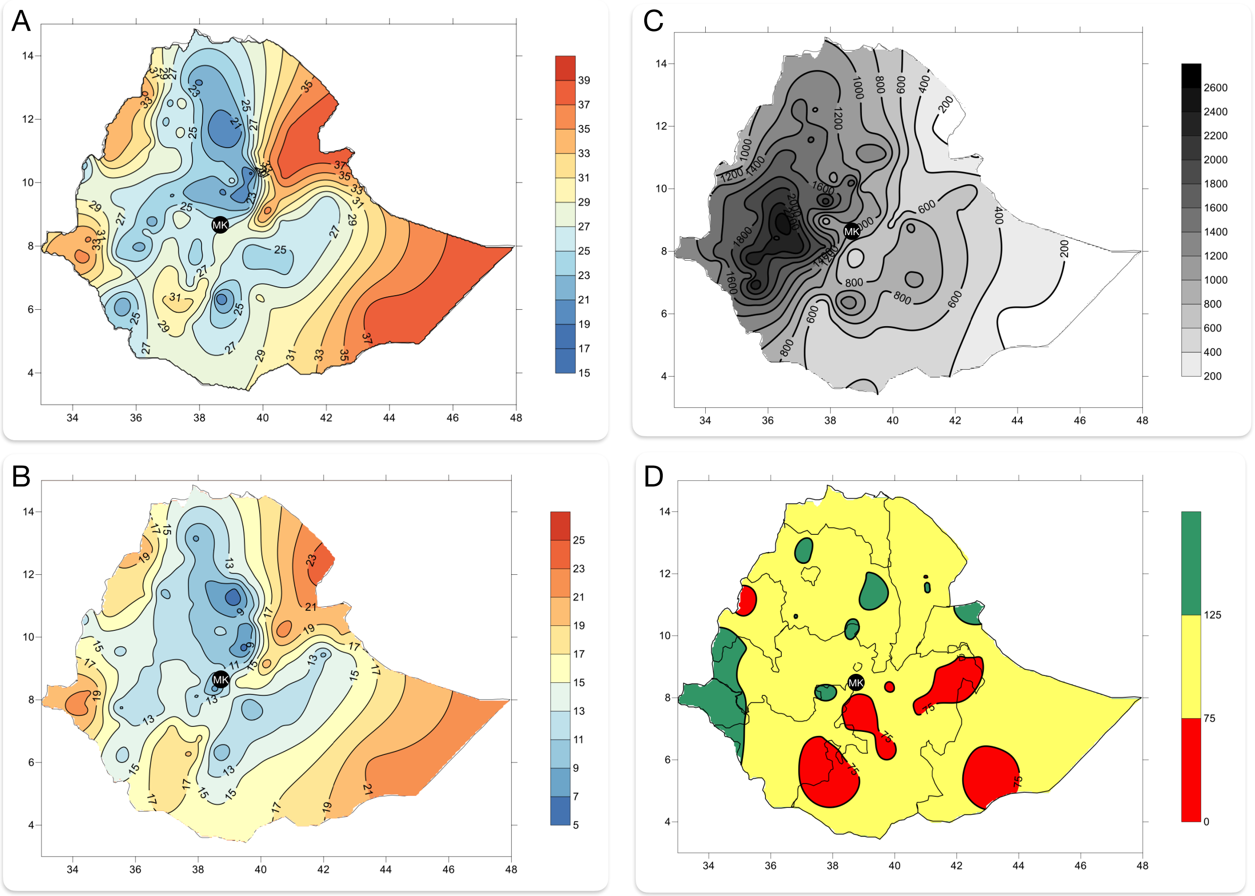

Figure S2. Mean maximum (A) and minimum (B) temperatures in °C, annual total rainfall amount in mm (C), and percent of normal rainfall (D) for the year 2022 (modified from Annual Climate Bulletin[2]). Black circle indicates the MK (Melka Kunture) localization.

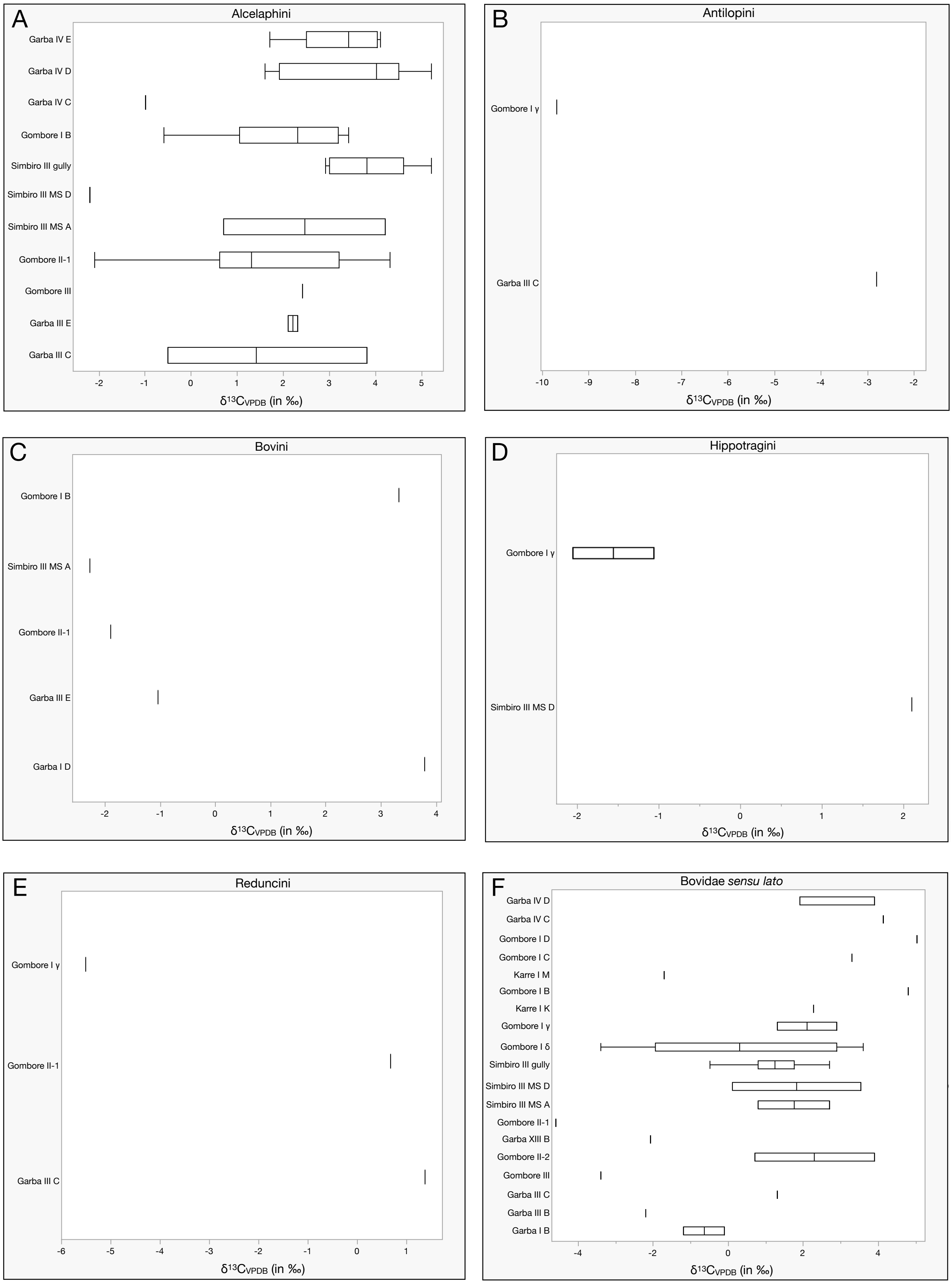

Figure. S3. Box and whisker plots of δ^{13}C values of Bovidae (Alcelaphini, Antilopini, Bovini, Hippotragini, Reduncini, and Bovidae *sensu lato*) from the MK archaeological sites (2.02 - 0.6 Ma). The vertical line in the boxes marks the median values, the box ends are

the lower and upper quartiles, and the lines define the range of data. A vertical line is equivalent to a dot/value.

Supplementary A. Stable carbon and oxygen isotopes in tooth enamel

The analysis of stable carbon and oxygen isotopic abundances in tooth enamel provides direct evidence of the dietary patterns, ecology, and habitat since the isotopic signal is related to the plants consumed and the water ingested during the formation of the analyzed tissue. Tooth enamel is the most suitable fossilized material for preserving stable isotopic signatures since it is almost entirely inorganic, less susceptible to alteration due to diagenesis, and its isotopic composition can be preserved over millions of years[3,4]. The stable isotopic results are typically reported in the common δ-notation: $X = [(R_{sample} / R_{standard}) - 1] * 1000$, where X is referred to as $\delta^{13}C$ and $\delta^{18}O$ values, and R represents $^{13}C/^{12}C$ or $^{18}O/^{16}O$, respectively.

The stable carbon isotope ratio in herbivore tooth enamel reflects an average diet related to the plants eaten by the animals during the period of enamel mineralization. Terrestrial plants are generally divided into C_3 and C_4 plants according to their different photosynthesis pathways, leading to different carbon isotopic fractionation during CO_2 fixation processes[5]. The C_3 photosynthetic pathway (Calvin-Benson cycle) occurs in trees, bushes, grasses in humid areas, and high-elevation herbaceous monocots[6,7]. In contrast, most tropical grasses, sedges, and some shrubs in dry/warm environments (e.g., Amaranthaceae) use the C_4 photosynthetic pathway (Hatch-Slack cycle). In modern-day Africa, the $\delta^{13}C$ value of C_4 plants ranges from - 19 ‰ to - 9 ‰, showing a modal value of - 13 ‰, whereas C_3 plants have a modal $\delta^{13}C$ value of - 27 ‰, ranging from - 35 ‰ to - 22 ‰[8,9]. Isotopic fractionation from diet to tooth takes place during enamel mineralization. During the last decades, a single diet-enamel enrichment value (+ 14 ‰) has been used[10]. However, Tejada-Lara et al.[11] found a significant correlation between body mass and isotope enrichment in herbivore mammals. According to Cerling and Harris[10] and Uno et al.[12], browsers have $\delta^{13}C_{enamel}$ values lower than - 8 ‰, grazers have values above - 2 ‰, and values in between are typical for mixed-feeders. Instead, carnivore $\delta^{13}C_{enamel}$ values reflect the isotopic signatures of their prey[13]. The abundance of C_4 plants suggests a relatively open grassy environment, whereas C_3 plants point to more woody vegetation under more humid climatic conditions[10,14].

The stable oxygen isotope composition in tooth enamel reflects the isotopic composition of the body water, which is determined by habitat, climate, diet, drinking behavior, and physiology[15,16]. In Africa, mammals are generally divided into two groups: evaporation sensitive (ES) and evaporation insensitive (EI). ES taxa (e.g., giraffids, oryx, dik-dik, gazelle, and buffalo) are those that ingest evaporated waters, showing a significant increase in $\delta^{18}O$ values between tooth enamel and meteoric water as a response to increasing aridity. In contrast, EI taxa (e.g., hippopotamus, bush pig, elephant, rhinoceros, warthog, zebra, impala, and baboon) ingest relatively unevaporated waters and show a strong correlation between enamel and meteoric water $\delta^{18}O$ values[17].

Due to evaporation, the $\delta^{18}O$ values in plant leaves are higher than those in meteoric water. This means that herbivores that get most of their water from consumed leaves have higher oxygen isotopic values than those drinking abundant meteoric water[15,18], allowing us to

distinguish browsers from grazers. Thus, $\delta^{18}O$ values will enable us to determine the so-called “obligate drinkers,” who obtain water from the rivers or lakes ($\delta^{18}O$ of meteoric water), from the other “non-obligate drinkers” that get most of their water from leaves, showing higher $\delta^{18}O$ values. The oxygen isotopic composition can also be affected by habitat differences: for semiaquatic mammals such as hippos, the $\delta^{18}O$ values are lower than those of terrestrial herbivores[19-22]. Furthermore, many other aspects can play a relevant role in the variation in oxygen isotope composition, such as precipitation, continentality, seasonality, latitude, and altitude[16].

Supplementary B. Laboratory activities

Faunal specimens. We selected well-preserved teeth and fragments (n = 308) of Hippopotamidae, Bovidae, Equidae, Suidae, Giraffidae, and Hyaenidae (Fig. S4) from the faunal assemblage of MK complex of sites to measure the $^{13}C/^{12}C$ and $^{18}O/^{16}O$ isotopic ratios. Fossil teeth, which were taxonomically identified and previously discussed by one of us[1,23], were unearthed from several archaeological layers of the following localities (from the oldest to the youngest): Karre I, Garba IV, Gombore I, Simbiro III, Garba XII, Garba XIII, Gombore II, Garba III, Garba I, and Gombore III (Fig. 1C).

Sampling. The enamel samples were collected in November 2018, April, July and November 2019, June and July 2022 at the National Museum of Ethiopia (Addis Ababa), in agreement with the Ethiopian Heritage Authority (EHA). The lab code corresponds to the abbreviation of the main site name (MLK = Melka Kunture), followed by a progressive number (i.e., MLK 1, MLK 2, MLK 3, etc.). The uppermost surface of the tooth was first cleaned with a diamond drill bit (> 2.0 mm) to remove potential contaminants attached to the enamel surface. When possible, enamel samples were collected along the length of the tooth (bulk sampling) to obtain an average diet and ecological setting during the period of tooth formation[24]. However, due to the fragmented status of some tooth remains, the internal fractures of enamel, and the concretions on the tooth surface, it was not always possible to encompass the full crown height of the archaeological teeth. As a result, some samples do not represent the full mineralization time of the tooth but rather a short period. Approximately 12 - 15 mg of enamel powder was obtained using a drilling device equipped with a diamond-tipped bit (< 2.0 mm). After each sample, the tools were cleaned with an ethanol solution to prevent contamination.

Pretreatment. This lab activity was conducted at the Biogeology Research Group of the University of Tübingen (Institut für Geowissenschaften, Hölderlinstrasse 12, Germany). The powdered enamel was soaked in 2 - 3 % NaOCl for 24 hours at 20 °C to oxidize organic residues and rinsed three times with Millipore water (Milli-Q H_2O) to remove all NaOCl. The remaining samples were treated with 0.1 M acetic acid-calcium acetate buffer (pH = 4.66) for 24 hours at 20 °C to remove exogenous carbonate. Subsequently, the samples were rinsed three times with Milli-Q H_2O and placed in an oven to dry at 40 °C for 72 hours. This pretreatment method is greatly used for cleaning bioapatite samples and effectively removes organic matter and exogenous carbonates. Only 2.5 - 3 mg of structural carbonate were subjected to Isotopic Ratio Mass Spectrometry (IRMS)[19,25-28].

Isotopic analyses. The pretreated samples were reacted with 99% phosphoric acid (H_3PO_4) for 4 hours at 70°C, using continuous-flow isotope ratio mass spectrometry (IRMS)

at the Biogeology Research Group. LyticOS software by Elementar was used to carry out multi-point standard isotope calibration by generating a trend line (y = mx + c) that maps measured versus expected isotopic results of standards, which was then used to calibrate sample results. The measurement uncertainty was monitored using three in-house standards. Two internal enamel samples used as secondary reference material (SRM) (Elephant and Hippo) were processed with each set of samples following the same protocol. Two international standards (IAEA-603, NBS-18) and one internal standard (LM = Laaser Marmor) were added to the data set (every 15 samples), and these standards were not pretreated prior to analysis. Overall analytical precision is higher than 0.1‰ for carbon and better than 0.2‰ for oxygen isotopic values. The carbon isotope compositions are expressed relative to the Vienna PeeDee Belemnite (VPDB) standard, whereas the oxygen isotope composition is expressed relative to the Vienna Standard Mean Ocean Water (VSMOW).

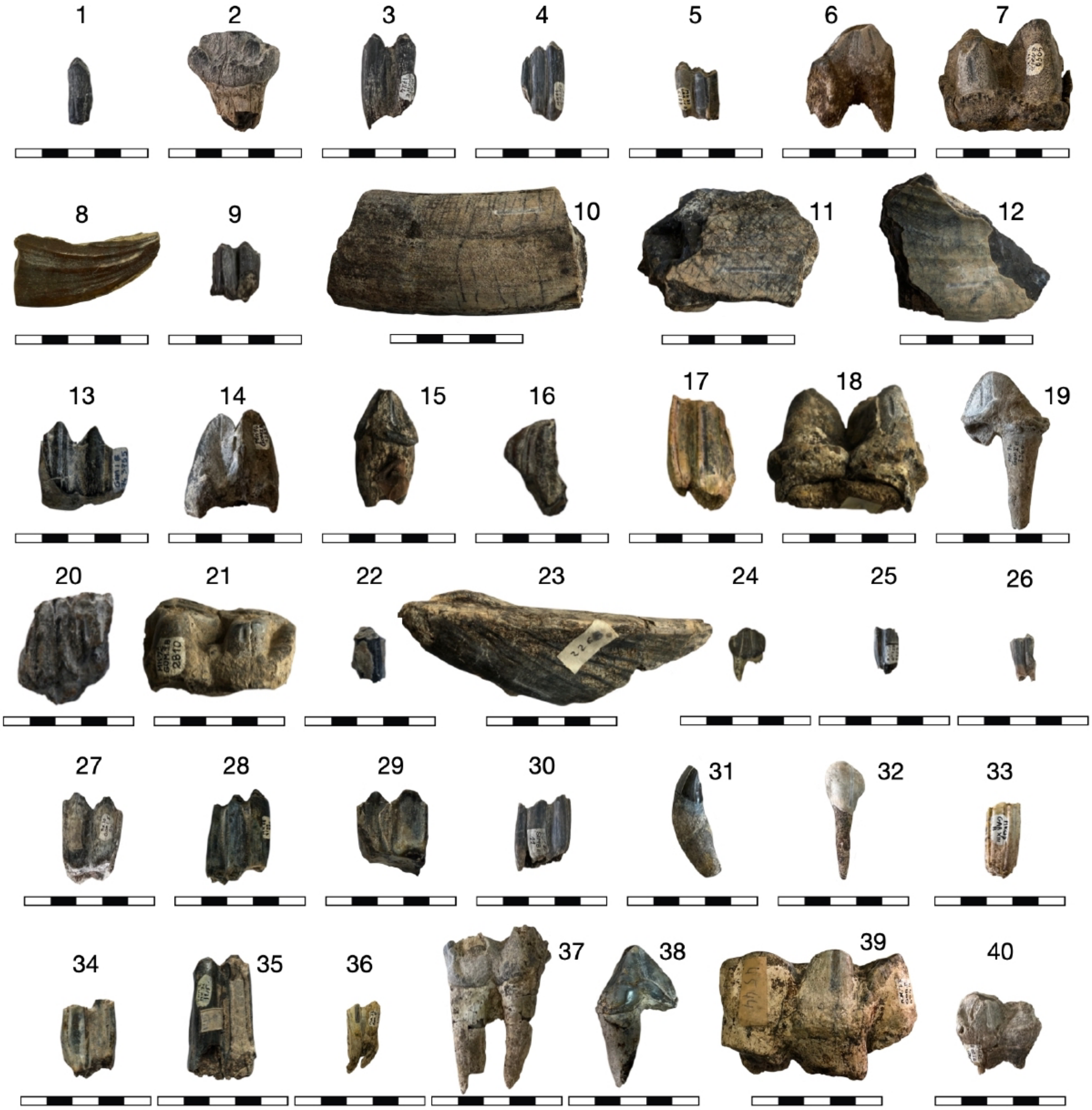

Figure S4. Examples of teeth sampled for the isotopic analyses: **1** Bovidae molar fragment (MK 80 KAR IM 143); **2** Hippopotamidae (*Hippopotamus* cf. *amphibius*) lower premolar (MK 04 GAR IVF178); **3** Bovidae (Alcelaphini) lower molar (MK 09 GAR IVE 1724); **4** Bovidae (Alcelaphini) lower molar (MK 74 GAR IVD 6561); **5** Bovidae (Alcelaphini) lower molar (MK 77 GAR IVD 5399); **6** Hippopotamidae (*Hippopotamus* cf. *amphibius*) upper premolar (MK 75 GAR IVD 632); **7** Hippopotamidae (*Hippopotamus* cf. *amphibius*) upper molar (MK 74 GAR IVD 6365); **8** Suidae (cf. *Metridiochoerus* sp.) upper canine fragment (MK 74 GAR IVD 6958); **9** Bovidae (Alcelaphini) lower molar (MK 77 GAR IVD 8250); **10** Hippopotamidae (*Hippopotamus* cf. *amphibius*) upper canine fragment (MK 74 GAR IVD 6394); **11** Hippopotamidae (*Hippopotamus* cf. *amphibius*) lower canine fragment (MK 74 GAR IVD 1230); **12** Hippopotamidae (*Hippopotamus* cf. *amphibius*) lower canine fragment (MK 75 GAR IVD 6750); **13** Bovidae (cf. Bovini) molar (MK 74 GOM IB 3755); **14** Hippopotamidae (*Hippopotamus* cf. *amphibius*) lower molar (MK 74 GOM IB 3285); **15** Hippopotamidae (*Hippopotamus* cf. *amphibius*) premolar (MK 74 GOM IB 90); **16** Suidae (*Metridiochoerus*) molar fragment (MK 74 GOM IB 3637); **17** Bovidae (Alcelaphini) upper molar (MK 73 GOM IB 4804); **18** Hippopotamidae (*Hippopotamus* cf. *amphibius*) upper molar (MK 74 GOM IB 5056); **19** Bovidae lower premolar (MK 74 GOM IB 3240); **20** Suidae (*Metridiochoerus*) molar fragment (MK 73 GOM IB 1487); **21** Hippopotamidae (*Hippopotamus* cf. *amphibius*) upper molar (MK 72 GOM IB 2810); **22** Bovidae (Alcelaphini) lower molar fragment (MK 70 GOM IB 1005); **23** Hippopotamidae (*Hippopotamus* cf. *amphibius*) upper canine fragment (MK 69 GOM IB 2268); **24** Suidae (*Kolpochoerus*) upper premolar (MK 81 GOM IB 9400); **25** Bovidae (Alcelaphini) lower molar (MK 73 GOM IB 6623); **26** Bovidae upper molar (MK 74 GOM Iγ 125); **27** Bovidae (Hippotragini, *Oryx*) lower molar (MK 70 GOM Iγ 943); **28** Bovidae (Alcelaphini) lower molar (MK 68 SIM III MS A 376); **29** Bovidae (Hippotragini) upper molar (MK 74 SIM III MS D 104); **30** Bovidae (Alcelaphini) lower molar (MK 71 SIM III MS D 11); **31** Hyaenidae lower canine (MK 18 SIM III gully 293); **32** Bovidae incisor (MK 07 GAR XIII A); **33** Equidae lower molar (MK 07 GAR XIII B); **34** Equidae lower molar (MK 75 GOM II-1 751); **35** Bovidae (Alcelaphini, *Connochaetes*) upper molar (MK 76 GOM II-1 1215); **36** Bovidae (Alcelaphini) lower premolar (MK 73 GOM II-1 4560); **37** Hippopotamidae (*Hippopotamus* cf. *amphibius*) lower premolar (MK 74 SIM III MS D 35); **38** Hippopotamidae (*Hippopotamus* cf. *amphibius*) lower premolar (MK 18 SIM III gully 322); **39** Hippopotamidae (*Hippopotamus* cf. *amphibius*) lower molar (MK 74 GOM II-1 4544); **40** Hippopotamidae (*Hippopotamus* cf. *amphibius*) lower premolar (MK 75 GAR IB 1785); scale bar = 5 cm.

Supplementary C. Isotopic comparison of Early and Middle Pleistocene fauna

We gathered 753 isotopic values ($\delta^{13}C$ and $\delta^{18}O$) of fauna tooth enamel from published data of eastern African sites at medium and low altitudes (≤ 1500 m a.s.l.), such as Olduvai Gorge, Lake Turkana Basin, and Busidima Formation, to evaluate whether the differences in elevation and vegetation composition were an influencing factor on animal feeding strategies and their habitats. We discuss 1.064 isotopic values, including data from MK (n = 308) presented in this study. This isotopic dataset includes six faunal families (Hippopotamidae, Bovidae, Equidae, Suidae, Giraffidae, and Hyaenidae) dated between 2.1 and 0.6 Ma (Tab. S4).

Hippopotamidae. Hippos (n = 239) from Lake Turkana Basin, Olduvai Gorge, Busidima Formation, and MK belong to several hippo species. The median $\delta^{13}C$ value is - 0.4 ‰ with a range from - 8.7 ‰ to + 2.8 ‰. The $\delta^{18}O$ values show a median of + 25.2 ‰ with a range from + 18.2 ‰ to + 38 ‰ (Figs. S5A - S6A, Tabs. S5 – S6).

Bovidae. Bovids (n = 479) from Lake Turkana Basin, Olduvai Gorge, Busidima Formation, and MK belong to several bovid tribes and species. The median $\delta^{13}C$ value is + 0.5 ‰ with a range from - 13.4 ‰ to + 5.2 ‰. The $\delta^{18}O$ values show a median of + 30 ‰ with a range from + 18.2 ‰ to + 37 ‰ (Figs. S5B - S6B, Tabs. S5 - S6).

Equidae. Equids (n = 135) from Lake Turkana Basin, Olduvai Gorge, Busidima Formation, and MK belong to several equid species. The median $\delta^{13}C$ value is + 0.4 ‰ with a range from - 7.6 ‰ to + 6.4 ‰. The $\delta^{18}O$ values show a median of + 29.4 ‰ with a range from + 23.1 ‰ to + 35 ‰ (Figs. S5C - S6C, Tabs. S5 - S6).

Suidae. Suids (n = 164) from Lake Turkana Basin, Olduvai Gorge, Busidima Formation, and MK belong to several suid species. The median $\delta^{13}C$ value is - 0.5 ‰ with a range from - 6.7 ‰ to + 3.3 ‰. The $\delta^{18}O$ values show a median of + 29.5 ‰ with a range from + 20.1 ‰ to + 37 ‰ (Figs. S5D - S6D, Tabs. S5 - S6).

Hyaenidae. Hyaenas (n = 14) from Olduvai Gorge and MK include *Crocuta* sp. and other hyaenas not identified. The average $\delta^{13}C$ value is - 1.3 ‰ with a range from - 4.2 ‰ to + 2.5 ‰. The $\delta^{18}O$ values show an average of + 26.3 ‰ with a range from + 22.7 ‰ to + 31.3 ‰ (Figs. S5E - S6E, Tabs. S5 - S6).

Giraffidae. Giraffids (n = 28) from Lake Turkana Basin, Olduvai Gorge, Busidima Formation, and MK belong to several giraffid species. The average $\delta^{13}C$ value is - 6.6 ‰ with a range from - 14.7 ‰ to + 2.7 ‰. The $\delta^{18}O$ values show an average of + 31 ‰ with a range from + 24.5 ‰ to + 39.5 ‰ (Figs. S5F- S6F, Tabs. S5 - S6).

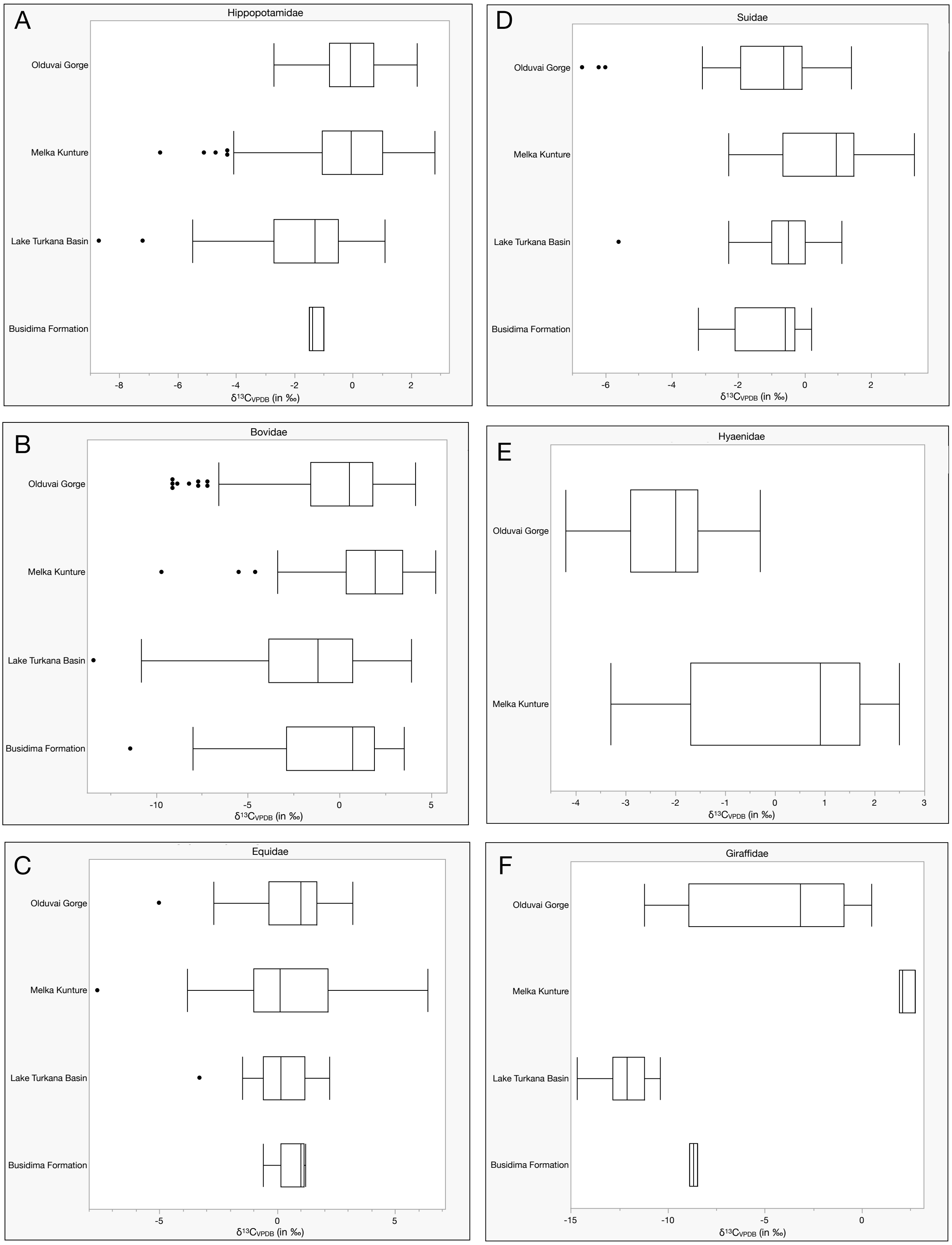

Figure. S5. Box and whisker plots of δ^{13}C values of hippos (A), bovids (B), equids (C), suids (D), hyenas (E), and giraffids (F) from Lake Turkana Basin, MK, Olduvai Gorge, and Busidima Formation. The vertical line in the boxes marks the median values; the box ends are the lower and upper quartiles; the lines define the range of data; a single dot is equivalent to a value.

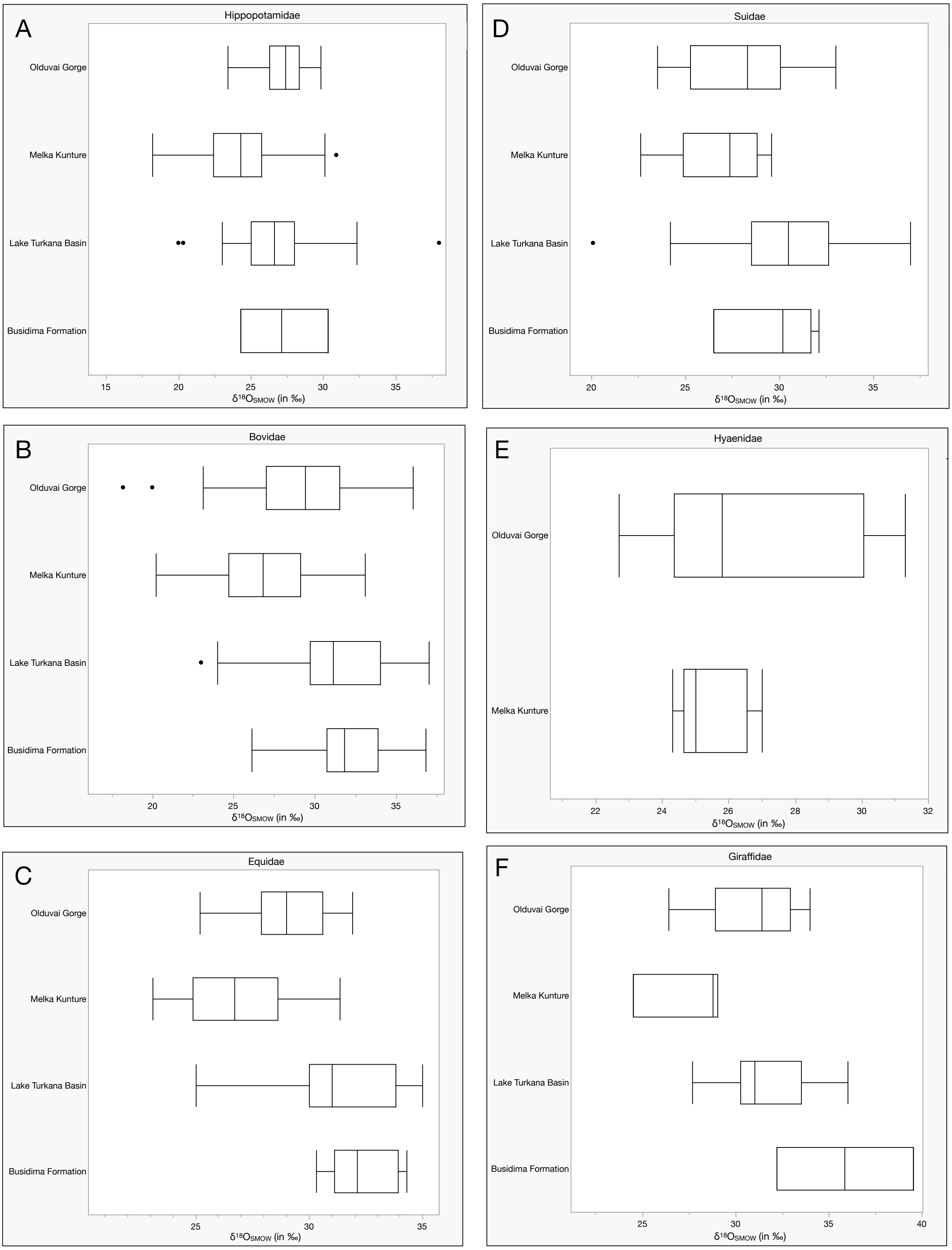

Figure. S6. Box and whisker plots of $\delta^{18}O$ values of hippos (A), bovids (B), equids (C), suids (D), hyenas (E), and giraffids (F) from Lake Turkana Basin, MK, Olduvai Gorge, and Busidima Formation. The vertical line in the boxes marks the median values; the box ends are the lower and upper quartiles; the lines define the range of data; a single dot is equivalent to a value.

References

1. Geraads, D., Eisenmann, V., Petter, G. The large mammal fauna of the Oldowan sites of Melka Kunture. In: Chavaillon, J. and Piperno, M. (Eds), *Studies on the Early Paleolithic site of Melka Kunture, Ethiopia*. Origines, Istituto Italiano di Preistoria e Protostoria, 169-192 (2004).
2. Ethiopian Meteorological Institute – Data and climatology directorate (Federal Democratic Republic of Ethiopia, Ministry of water and energy). Annual climate bulletin for the year 2022. Available at: www.ethiomet.gov.et (2022).
3. Wang, Y., Cerling, T.E. A model of fossil tooth and bone diagenesis: Implications for paleodiet reconstruction from stable isotopes. *Palaeogeogr., Palaeoclimatol., Palaeoecol.* **107**, 281-289, https://doi.org/10.1016/0031-0182(94)90100-7 (1994).
4. Schoeninger, M.J., Hallin, K., Reeser, H., Valley, J.W., Fournelle, J. Isotopic alteration of mammalian tooth enamel. *Int. J. Osteoarchaeol.* **13**, 11-19, https://doi.org/10.1002/oa.653 (2003).
5. Edwards, G., Walker, D.A. C_3, C_4: Mechanisms, and Cellular and Environmental Regulation, of Photosynthesis. *Blackwell Scientific Publications*, Oxford, Vol.6, No.11 (1983).
6. Vogel, J.C. Isotopic assessment of the dietary habits of ungulates. *S. Afr. J. Sci.* **74**, 298–301 (1978).
7. Blondel, C., *et al*. Feeding ecology of Tragelaphini (Bovidae) from Shungura Formation, Omo Valley, Ethiopia: Contribution of dental wear analyses. *Palaeogeogr., Palaeoclimatol., Palaeoecol.* **496**, 103-120, https://doi.org/10.1016/j.palaeo.2018.01.027 (2018).
8. Smith, B.N., Epstein, S. Two categories of $^{13}C/^{12}C$ ratios for higher plants. *Plant Physiol.* **47**, 380-384, 10.1104/pp.47.3.380 (1971).
9. O'Leary, M.H. Carbon Isotopes in Photosynthesis. *BioScience* **38**, 328-336, https://doi.org/10.2307/1310735 (1988).
10. Cerling, T.E., Harris, J.M. Carbon Isotope Fractionation between Diet and Bioapatite in Ungulate Mammals and Implications for Ecological and Paleoecological Studies. *Oecologia* **120**, 347-363, https://doi.org/10.1007/s004420050868 (1999).
11. Tejada-Lara, J.V., *et al*. Body mass predicts isotope enrichment in herbivorous mammals. *Proc. Biol. Sci.* **285**, 20181020, doi:10.1098/rspb.2018.1020 (2018).
12. Uno, K.T., *et al*. Large mammal diets and paleoecology across the Oldowan-Acheulean transition at Olduvai Gorge, Tanzania from stable isotope and tooth wear analyses. *J. Hum. Evol.* **120**, 1-16, https://doi.org/10.1016/j.jhevol.2018.01.002 (2018).
13. Bocherens, H., Drucker, D. Trophic level isotopic enrichment of carbon and nitrogen in bone collagen: Case studies from recent and ancient terrestrial ecosystems. *Int. J. Osteoarchaeol.* **13**, 46–53, https://doi.org/10.1002/oa.662 (2013).
14. Farquhar, G.D., Ehleringer, J.R., Hubick, K.T. Carbon Isotope Discrimination and Photosynthesis. *Annu. Rev. Plant Biol.* **40**, 503-537, https://doi.org/10.1146/annurev.pp.40.060189.002443 (1989).
15. Kohn, M.J. Predicting animal ^{18}O: accounting for diet and physiological adaptation. *Geochim. Cosmochim. Acta* **60**, 4811-4829, https://doi.org/10.1016/S0016-7037(96)00240-2 (1996).

16. Pederzani, S., Britton, K. Oxygen Isotopes in Bioarchaeology: Principles and Applications, Challenges and Opportunities. *Earth-Sci. Rev.* **188**, 77-107, https://doi.org/10.1016/j.earscirev.2018.11.005 (2019).
17. Levin, N.E., Cerling, T.E., Passey, B.H., Harris, J.M., Ehleringer, J.R. A stable isotope aridity index for terrestrial environments. *PNAS* **103** (30), 11201-11205, www.pnas.orgcgidoi10.1073pnas.0604719103 (2006).
18. Kohn, M.J., Schoeninger, M.J., Valley, J.W. Variability in oxygen isotope compositions of herbivore teeth. *Chem. Geol.* **152**, 97-112, https://doi.org/10.1016/S0009-2541(98)00099-0 (1998).
19. Bocherens, H., Koch, P.L., Mariotti, A., Geraads, D., Jaeger, J.-J. Isotopic biogeochemistry (^{13}C, ^{18}O) of mammalian enamel from African Pleistocene hominid sites. *Palaios* **11**, 306-318, https://doi.org/10.2307/3515241 (1996).
20. Clementz, M.T., Koch, P.L. Differentiating aquatic mammal habitat and foraging ecology with stable isotopes in tooth enamel. *Oecologia* **129**, 461-472, https://doi.org/10.1007/s004420100745 (2001).
21. Clementz, M.T., Holroyd, P.A., Koch, P.L. Identifying aquatic habits of herbivorous mammals through stable isotope analysis. *Palaios* **23** (9), 574-585, https://doi.org/10.2110/palo.2007.p07-054r (2008).
22. Harris, J.M., Cerling, T.E., Leakey, M.G., Passey, B.H. Stable isotope ecology of fossil hippopotamids from the Lake Turkana Basin of East Africa. *J. Zool.* **275**, 323-331, https://doi.org/10.1111/j.1469-7998.2008.00444.x (2008).
23. Geraads, D., Gallotti, R., Raynal, J.-P., Bonnefille, R., Mussi, M. Melka Kunture, Ethiopia: Early Pleistocene Faunas of the Ethiopian Highlands. In: Reynolds, S.C., Bobe, R. (eds) *African Paleoecology and Human Evolution*. Cambridge University Press, Cambridge, 256-268 (2022).
24. Fricke, H.C., O'Neil, J. Inter-and intra-tooth variation in the oxygen isotope composition of mammalian tooth enamel phosphate: implications for palaeoclimatological and palaeobiological research. *Palaeogeogr., Palaeoclimatol., Palaeoecol.* **126**, 91-99, https://doi.org/10.1016/S0031-0182(96)00072-7 (1996).
25. Koch, P.L., Tuross, N., Fogel, M.L. The effects of sample treatment and diagenesis on the isotopic integrity of carbonate in biogenic hydroxylapatite. *J. Archaeol. Sci.* **24**, 417-429, https://doi.org/10.1006/jasc.1996.0126 (1997).
26. Snoeck, C., Pellegrini, M. Comparing bioapatite carbonate pre-treatments for isotopic measurements: Part 1 - Impact on structure and chemical composition. *Chem. Geol.* **417**, 394-403, http://dx.doi.org/10.1016/j.chemgeo.2015.10.004 (2015).
27. Pellegrini, M., Snoeck, C. Comparing bioapatite carbonate pre-treatments for isotopic measurements: Part 2 - Impact on carbon and oxygen isotope compositions. *Chem. Geol.* **420**, 88-96, http://dx.doi.org/10.1016/j.chemgeo.2015.10.038 (2016).
28. Wright, L.E., Schwarcz, H.P. Correspondence between stable carbon, oxygen and nitrogen isotopes in human tooth enamel and dentine: infant diets at Kaminaljuyu. *J. Archaeological. Sci.* **26**, 1159-1173, https://doi.org/10.1006/jasc.1998.0351 (1999).
